# 

**Published:** 1910-05-28

**Authors:** 


### APPOINTMENTS.

At a meeting of the General Court of Governors of Guy's Hospital, Mr. W. M. Mollison, M.C., F.R.C.S., was appointed Aural Surgeon, and Mr. A. R. Thompson, Ch.M., F.R.C.S., surgeon to the newly established genito-urinary department of the hospital.

The following appointments on the Honorary Medical Staff of King's College Hospital have been made: Dr. Urban Pritchard, to be consulting aural surgeon; Mr. Arthur H. Cheatle, F.R.C.S., to be lecturer in and teacher of aural surgery; and Mr. G. J. Jenkins, F.R.C.S., to be assistant aural surgeon.

At the meeting of the Board of the Leeds General Infirmary, held on Friday, May 6, Mr. Matthew J. Stewart, M.B., Ch.B., Glasgow, was appointed to the position of clinical pathologist to the infirmary, recently vacated by Dr. O. C. Grüner on his appointment to the McGill University and Royal Victoria Hospital, Montreal.

The Local Government Board for Scotland has made the following appointments to the additional posts which have been created on their staff—namely, Dr. Thomas F. Dewar, M.D., C.M., D.Sc., presently M.O.H. for the counties of Fife and Kinross, to be a medical inspector; Miss Elizabeth M. M'Vail, M.B., Ch.B.Glas., D.P.H.Camb., to be Lady Inspector.

### Gifts and Bequests.

Mr. Thomas Horne, of Solihull, who died on April 11, aged eighty-eight, left, after certain bequests, the residue of his property, subject to certain annuities, equally between the Royal National Lifeboat Institution, the Birmingham Eye Hospital, and the General Institution for the Blind, Edgbaston. It would appear that the total amount available under Mr. Horne's will for charitable purposes will be about £36,000.

The will of Sir Frederick Thorpe Mappin, formerly M.P. for the Hallamshire Division, has been proved at £931,086. He left Sheffield University, Sheffield Royal Infirmary, Sheffield Royal Hospital, Jessop Hospital, Sheffield, and the Royal Albert Asylum, Lancaster, each one thousand pounds.

Mrs. Kate Jacobs, of Mapesbury Road, Cricklewood, left £100 each to the Jewish Soup Kitchen, the Jewish Orphan Asylum, Norwood, the Jewish Home for the Deaf and Dumb, the Jewish Home for Incurables, and the Jewish Home for the Blind.

Mrs. Elizabeth Hannah Gartside, of Upper Brook Street, W., left £100 to the Hospital for Sick Children, Great Ormond Street.

The Leicester Infirmary has received a donation of £25 from the Trustees of Henry Smith's (Longney Manor, etc., estates) Charity, per R. A. Warren, Esq., Treasurer.

The North London Nursing Association, 413 Holloway Road, N., has received a grant of £20 from the Goldsmiths' Company.

In commemoration of his winning the One Thousand Guineas, Mr. Waldorf Astor, of Cliveden, has handed to the Mayor of Maidenhead £100 for the benefit of the poor of the town. With the consent of the donor, the Mayor proposes to buy presentations to the Metropolitan Convalescent Home, to be administered by the Mayor of Maidenhead for the time being.

Mr. Alfred John Bowman, of Tunbridge Wells, barrister, has left £100 each to ten friends, "to invest in port wine or anything else they like" in memory of him, and £100 to the Tunbridge Wells General Hospital.

The Rev. Slade Baker-Stallard-Penoyre, of Edenholme, Cheltenham, has bequeathed £20 to the Kidderminster Hospital.

Miss Elinor Stewart, of North Kensington, has left £100 to the Homœopathic Hospital, London.

Among the contributions received "In Memoriam King Edward VII." by his late Majesty's Hospital Fund are Mr. D. Q. Henriques, £50; "Anonymous," £50; Marlborough College Offertory, £13 11s. 6d.; The Constable, the Lieutenant, and the Major of the Tower of London, £10; Mrs. C. A. Arnold, £10; Wellington College Offertory, £9 11s. 6d.; A poor lady, 2s. 6d.

St. Mary's Hospital acknowledges the following donations: from the Worshipful Company of Goldsmiths, £500; Miss Tidswell, £100; the Worshipful Company of Skinners, £40.

The Royal Westminster Ophthalmic Hospital has received £65 from the trustees of Smith's (Kensington Estate) Charity.

The Chelsea Hospital for Women has received £270 from the trustees of Smith's (Kensington Estate) Charity and also £50 from the Goldsmiths' Company.

Mrs. Winifred Crew Wing, of South Kensington, has left £500 to the Eversfield Chest and Throat Hospital, St. Leonards.

### Jottings.

The King of the Belgians, who, like his two predecessors, has become a patron of the Finsbury Dispensary, and whose health was drunk at the recent biennial festival, has caused the following telegram to be sent to the Lord Mayor, who presided: "His Majesty, deeply touched by your good wishes, has entrusted me to send you his best thanks and compliments, and to ask you to convey them also in his name to the Sheriffs of London and the governors and friends of the Finsbury Dispensary.—Baron Beyens, Minister of the King's Household."

The Saturday and Sunday Funds for New York for the year 1909 were apportioned among the various charity hospitals of New York City at a meeting held in Mayor Gaynor's office recently. The total amount of the funds for the year is $76,000. The largest sums, which are apportioned according to the amount of free work done at each hospital, were given to the Montefiore Home, and Mount Sinai Hospital, which received $7,600 and $7,551, respectively.

A sum of $179,000 has been voted for the beginning of construction of Letchworth Village, the new State institution at Thiells for the care of epileptics and idiots.

Her Majesty the Queen has sent again from Sandringham a gift of flowers to be distributed to the invalid children under the care of the Invalid Children's Aid Association.

Lord Strathcona and Mount Royal will preside at the Festival Dinner, which will be held on Monday, June 20, at the Whitehall Rooms, in aid of the urgent appeal for funds on behalf of the Metropolitan Hospital, Kingsland Road, N.E.

Amid her many interests and sympathies, the work which Sir William Treloar has been doing for crippled boys and girls at Alton has always received the Queen-Mother's personal interest and support. The Juvenile League which is helping the home bears her name, "The Queen Alexandra League of Children to Help Poor Crippled Children." It now numbers over 12,000 collectors, and Sir William Treloar is making a strong appeal to them to raise £1,000 among themselves to permanently endow a cot in the home—"The King Edward VII. Memorial Cot"—as a token of their sympathy with their president in her great grief and of their deep sorrow at the loss of King Edward, who also took great interest in this work for crippled children.

A meeting of the Victoria Hospital, Swindon, Management Committee has been held, Mr. H. Kinneir presiding, when thanks were heartily given to Mr. Alfred Manners for his great kindness in promoting again the sacred concert in aid of the hospital. This concert took place at the Empire Theatre, Swindon, and all the expenses were borne by Mr. Manners.

The annual sale of inmates' work of the Royal Hospital for Incurables will be held at the Hospital, West Hill, Putney, June 7, 8, and 9. The Most Hon. the Marchioness of Salisbury will open it on June 7, at 2.30 p.m.

Mr. J. F. Mason, M.P. for Windsor, has sent the following letter to the Mayor: "I feel that on this day of national mourning I must ask you to hand the enclosed cheque for £500 to King Edward VII. Hospital at Windsor, in which institution his late Majesty took so keen an interest."

THE

### GRADUATES' MEDICAL SCHOOLS.

DIARY FOR THE WEEK, MAY 30 to JUNE 4.

**ROYAL SOCIETY OF MEDICINE,** 15 Cavendish Square, W. (Temporary address during building.)

At 5 p.m.—**May 31, Dermatological Section:**
At 11 Chandos Street, W.
*Demonstration* of cases of Lupus erythematosus.

[Note.—This is the meeting which was announced for May 13 and was postponed.]

5 p.m.—**June 1,** Special Meeting of Fellows of the Society.
At Morley Hall, George Street, Hanover Square, W.
*Discussion* on "Vaccine Therapy; its administration, value, and limitations" (third meeting). Re-opened by Dr. William Bulloch, and continued by Sir William B. Leishman, Sir John McFadyean, Professor R. T. Hewlett, Mr. K. W. Goadby, Dr. J. Henderson Smith, Dr. Arthur Whitfield, Dr. John Freeman.

5 p.m.—**June 3, Laryngological Section:**
*Cases and Specimens* will be shown by Dr. Dundas Grant, Dr. Jobson Horne, Mr. Seccombe Hett, Dr. Cathcart, and others.

8.30 p.m.—**June 3, Electro-Therapeutical Section:**
*Annual General Meeting:* Election of officers and Council for Session 1910-1911.
*Paper*: Dr. Joseph Bolton: "The relief of symptoms of prostatic obstruction by electrical treatment."
*Discussion* on "The relative value of various types of high tension transformer (including coil) used for the production of *x*-rays." Opened by Dr. Reginald Morton.

10 a.m.—**June 4, Otological Section:**
At 11 Chandos Street, W.
*Paper*: Dr. Wyatt Wingrave: "Notes on the pathology of cholesteatoma."
*Cases and Specimens*: Mr. A. L. Whitehead: Temporo-sphenoidal abscess; vomiting entirely absent. Dr. P. H. Abercrombie and Dr. Dan McKenzie: Hysterical deafness with active vestibular reactions. Dr. H. J. Davis: Deformity of both pinnæ resulting from perichondritis following double mastoid operation. Dr. Hunter Tod: (1) Exostosis of right external meatus; (2) A case in which the clinical symptoms simulated a cerebellar abscess; (3) Cholesteatoma or keratosis obturans of the external auditory canal. Mr. George Wilkinson: Two cases of cholesteatoma of unusual size. Dr. P. Watson Williams: Myelogenous sarcoma of the right temporal bone with extension through the external meatus, resembling an aural polypus. Mr. G. J. Jenkins: Fracture of the temporal bone.

**LONDON SCHOOL OF CLINICAL MEDICINE,** Seamen's Hospital, Greenwich, S.E.

At 2.15 p.m.—**June 1,** Dr. F. Taylor, **Tremors and Convulsive Movements.**
3.15 p.m.—**June 3,** Mr. McGavin, **The Surgery of Urethral Stricture.**

**THE HOSPITAL FOR SICK CHILDREN,** Great Ormond Street, W.C.

**June 2,** Dr. Hutchison, **Hysteria in Childhood.**

**NORTH-EAST LONDON POST-GRADUATE COLLEGE,** Prince of Wales's General Hospital, Tottenham, N.

**May 31,** Dr. G. N. Meachen, **Demonstration of Selected Skin Cases.**

**THE POST-GRADUATE COLLEGE,** West London Hospital, Hammersmith, W.

At 10 a.m.—**May 30** and **June 2,** Demonstrations by Surgical Registrar.
10 a.m.—**June 3,** Demonstration by Medical Registrar.
11.30 a.m.—**May 31, Demonstrations of Minor Operations.**
12 noon.—**May 30, Pathological Demonstration.**
12.15 p.m.—**June 1,** Dr. Grainger Stewart, **Practical Medicine.** Lectures at 5 p.m.
**May 30,** Mr. Dunn, **Ocular Symptoms in General Disease.**
**May 31,** Dr. Low, **Sleeping Sickness.**
**June 1,** Dr. Beddard, **Practical Medicine.** Lecture II.
**June 2,** Mr. Bidwell, **Surgery of Gastric Ulcer.**
**June 3,** Dr. Saunders, **Clinical Lecture with Cases.**

**MEDICAL GRADUATES' COLLEGE AND POLYCLINIC,** 22 Chenies Street, W.C.

At 4 p.m.—**May 30,** Dr. Willmott Evans, **Clinique (skin).**
5.15 p.m.—**May 30,** Dr. Rision Russell, **Diagnosis of Disseminated Sclerosis.**
4 p.m.—**May 31,** Dr. F. J. Wethered, **Clinique (medical).**
5.15 p.m.—**May 31,** Dr. Chas. Mercier, **Demonstration of Mental Cases.**
4 p.m.—**June 1,** Mr. A. H. Tubby, **Clinique (surgical).**
5.15 p.m.—**June 1,** Mr. W. H. Clayton Greene, **On the Treatment of Certain Fractures.**
4 p.m.—**June 2,** Dr. O. K. Williamson, **Clinique (medical).**
5.15 p.m.—**June 2,** Mr. Thomson Walker, **The Diagnosis and Treatment of Papilloma of the Bladder.**
4 p.m.—**June 3,** Mr. Claud Worth, **Clinique (eye).**

**CENTRAL LONDON THROAT AND EAR HOSPITAL,** Gray's Inn Road, W.C.

**May 31,** Dr. Atkinson, **Pharynx and Naso-pharynx.**
**June 3,** Dr. Abercrombie, **Clinical Cases.**

**NATIONAL HOSPITAL FOR THE PARALYSED AND EPILEPTIC,** Queen Square, Bloomsbury, W.C.

**May 31** and **June 3,** Dr. James Taylor, **Eye Signs and Symptoms in Nervous Disorders.**

**THE BROMPTON HOSPITAL FOR CONSUMPTION AND DISEASES OF THE CHEST,** Fulham Road, S.W.

**June 1,** Dr. Mitchell Bruce, **Aortic Incompetence and its Clinical Lessons.**

**ST. MARK'S HOSPITAL FOR DISEASES OF THE RECTUM,** City Road, E.C.

**June 1,** Mr. Gordon Watson, **Innocent Growths of the Rectum.**

**The Institution Library.**

"Burdett's Hospitals and Charities," 1910. (Net, post free.) ... ... ... ... ... ... ... ...10s. 11d.
"Hospital Expenditure: The Commissariat." For the use of Managers. ... ... ... ... ... 2s. 6d.
"The District Nursing Association; Hints on how to Start it." (Net, post free.) ... ... ... 1s. 1d.
"A Legal Handbook for the use of Hospital Authorities." .. ... ... ... ... ... ... ... 2s. 6d.
Ledgers, Charts, Case-papers, and every kind of Institutional Literature.

Of all booksellers or of The Scientific Press, Limited, 28 and 29 Southampton Street, Strand, London, W.C.

THE HOSPITAL. May 28, 1910.

*Name* ..............................................

*Address* ..............................................

..............................................................

This Coupon must accompany manuscript or contributions intended for The Hospital.

